# Automethylation of protein arginine methyltransferase 6 (PRMT6) regulates its stability and its anti-HIV-1 activity

**DOI:** 10.1186/1742-4690-10-73

**Published:** 2013-07-17

**Authors:** Diane N Singhroy, Thibault Mesplède, Arielle Sabbah, Peter K Quashie, Jean-Pierre Falgueyret, Mark A Wainberg

**Affiliations:** 1 McGill University AIDS Centre, Lady Davis for Medical Research, Jewish General Hospital, 3755 Cote Sainte Catherine, Montreal, QC, H3T 1E2, Canada; 2 Department of Microbiology and Immunology, McGill University, Montreal, QC, Canada; 3 Division of Experimental Medicine, Faculty of Medicine, McGill University, Montreal, QC, Canada; 4 Centre for Biological Application of Mass Spectrometry, Concordia University, Montreal, QC, Canada

**Keywords:** PRMT6, HIV-1, Automethylation, Protein stability, Antiretroviral activity

## Abstract

**Background:**

Protein arginine methyltransferase 6 (PRMT6) is a nuclear enzyme that methylates arginine residues on histones and transcription factors. In addition, PRMT6 inhibits HIV-1 replication in cell culture by directly methylating and interfering with the functions of several HIV-1 proteins, i.e. Tat, Rev and nucleocapsid (NC). PRMT6 also displays automethylation capacity but the role of this post-translational modification in its antiretroviral activity remains unknown.

**Results:**

Here we report the identification by liquid chromatography-mass spectrometry of R35 within PRMT6 as the target residue for automethylation and have confirmed this by site-directed mutagenesis and *in vitro* and *in vivo* methylation assays. We further show that automethylation at position 35 greatly affects PRMT6 stability and is indispensable for its antiretroviral activity, as demonstrated in HIV-1 single-cycle TZM-bl infectivity assays.

**Conclusion:**

These results show that PRMT6 automethylation plays a role in the stability of this protein and that this event is indispensible for its anti-HIV-1 activity.

## Background

Throughout its replication cycle, HIV interacts with a plethora of cellular proteins that can either restrict or aid infection. Recent genome-wide screens have identified many of the factors that contribute to HIV pathogenesis, in particular through the regulation of HIV protein function [1-6]. These interactions can sometimes result in post-translational modifications that are important in the regulation of HIV proteins. For example, acetylation of Tat regulates its transcriptional activity [7] and serine phosphorylation is essential for the activity of Vif [8].

Arginine methylation is a posttranslational modification in eukaryotes that results in the covalent addition of one or two methyl groups to the terminal nitrogen atom of arginine [9]. This reaction is catalyzed by protein arginine methyltransferases (PRMTs) and has been implicated in transcriptional regulation, epigenetics, DNA repair, mRNA splicing and signal transduction [10,11]. Arginine methylation is dependent on the methyl donor S-adenosyl-L-methionine (SAM) to yield the methylated arginine and an S-adenosylhomocysteine [12]. Eleven PRMTs have been characterized to date and are classified as type I, II or III [13]. Both type I and type II produce ω-N^G^-monomethylated arginine (MMA) intermediates, however type I enzymes further catalyze the formation of ω-N^G^- N^G^-asymmetric dimethylarginines (aDMA), while type II enzymes catalyze N^G^-N^G^-symmetric dimethylarginines (sDMA) [12,14]. Type III PRMTs only catalyze the formation of MMA residues. The addition of a methyl group does not change the overall charge of a protein, but can reallocate hydrogen bond sites, thus affecting protein-protein interactions [15]. The importance of arginine methylation in the cell is further supported by the fact that over 200 proteins contain putative dimethylated arginines [16].

PRMT6 is a 41.9 kDa Type I methyltransferase found in the nucleus [17]. It typically targets arginine in glycine and arginine rich (GAR) motifs. Thrombospondin-1 (TSP-1), H3R2 and H2A were all found to be arginine methylated by PRMT6 [18-20]. Our group has found that PRMT6 methylates and restricts the function of the HIV proteins Tat, Rev, and Nucleocapsid (NC) [21-24], resulting in restriction of HIV-1 replication by PRMT6. Methylation of Tat by PRMT6 leads to a disruption of the Tat-TAR-cyclin T1 complex and decreases Tat specific transcriptional activation [21]. Once NC is methylated by PRMT6, it is less able to promote RNA annealing and initiate reverse transcription [24].

The HIV-1 Rev protein is a 19 kDa protein found in the nucleolus, the perinuclear zone, and the cytoplasm of HIV infected cells. It mediates viral protein expression at the level of viral RNA splicing and nuclear export of unspliced and single spliced viral RNA by binding to the *cis*-acting Rev response element (RRE) [25,26]. Rev is arginine methylated by PRMT6 in its arginine rich motif (ARM) located within the Rev nuclear localization signal (NLS) [23]. Rev-mediated export of viral RNA is decreased when Rev is methylated by PRMT6 because methylated Rev is unable to bind efficiently to the RRE [23].

Automethylation of PMRT6 has been reported [17], but the site and role of automethylation have not been identified. PRMT6 requires homodimerization to transfer a methyl group from SAM to the protein substrate and this could favour automethylation [27]. As documented for CARM1 (PRMT4), another member of the PRMT family, we have hypothesized that automethylation could modulate PRMT6 function [28]. Mutagenesis of the automethylation site of CARMI did not affect its catalytic activity but impaired its transcriptional and RNA-processing capacity. Additionally, arginine methylation of the HIV protein Tat by PRMT6 and of the cellular protein axin by PRMT1 increase the stability of these proteins [29,30]. We now report that R35 is crucial for PRMT6 automethylation and that arginine automethylation is important for PRMT6 stability and its ability to inhibit HIV-1 replication.

## Results

### Identification of PRMT6 automethylation sites *in vitro* and *in vivo*

**Figure 1:**
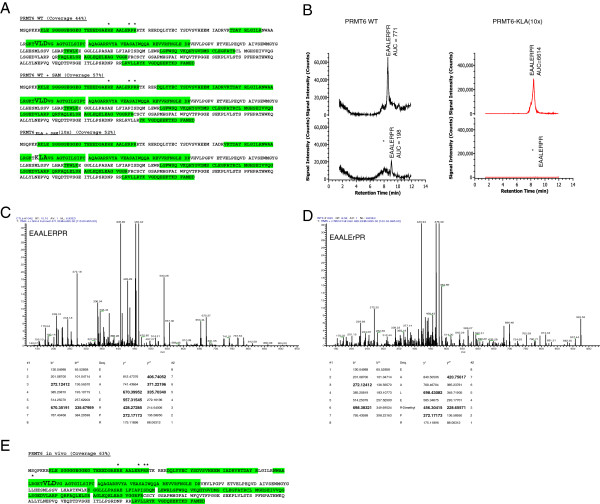
**Mass spectrometric analysis of PRMT6 arginine methylated residues.** Following *in vitro* and *in vivo* methylation, methylated arginine residues in recombinant PRMT6 were mapped by LC-MS/MS. **(A)** Percent coverage obtained for PRMT6 WT (−/+ SAM) and the PRMT6 KLA mutant. Recombinant PRMT6 was digested with Trypsin in an ammonium bicarbonate buffer and peptides were separated onto a C18 column and sequenced by LC-MS (Methods). To evaluate trace amounts of dimethylated arginine in mutated protein, a 10 fold concentrated peptide solution was injected. Percentages coverage for PRMT6, PRMT6 + SAM and the PRMT6 mutant were 44%, 57%, and 52%, respectively (highlighted in green). Dimethylated arginine was identified only with PRMT6 WT +/− SAM (R29; R35; R37). Dimethylated residues are represented with an (*) above the residue. **(B)** To evaluate the percentage of dimethylated arginine on PRMT6, ions corresponding to EAALERPR (m/z 471.26) and EAALER*PR (m/z 485.27) were extracted. Dimethylation was only observed in the WT protein and in the WT protein + SAM (not shown). There was no detectable signal at m/z 485.27 in the PRMT6 KLA mutant even when used at 10-fold the WT protein concentration. Areas under the curve (AUC) were studied for both peptides from the PRMT6 WT protein; assuming no differences in ionization efficiency, the methylated protein apparently represents 10 to 20% of total protein. MS/MS spectra observed for the methylated **(C)** EAALERPR and non-methylated **(D)** peptide of wild type PRMT6. Observed ions are indicated in bold. **(E)***In vivo* methylation assays were performed in HeLa cells with transfected myc-tagged PRMT6. Samples were digested and processed as described for the *in vitro* methylation assays. The percent coverage for PRMT6-WT was 63% (highlighted in green). Dimethylated arginines were identified for residues R29, R35, R38, R39 and R82, and are represented with an (*). Amino acids mutated in PRMT6-KLA are indicated by enlarged letters.

PRMT6 was previously shown to be automethylated [17]. Now, we wished to identify the arginine residues that are involved and, therefore, used purified recombinant PRMT6 in an *in vitro* methylation assay in the presence and absence of the methyl group donor S-adenosyl-methionine (SAM) with catalytically inactive recombinant PRMT6 V86K/D88A (PRMT6-KLA) serving as a control. Post-translational methylation was determined by mass spectrometry and showed that R29, R35 and R37 in the N-terminal region of the protein were methylated in the PRMT6 wild-type protein with and without SAM but not in the KLA inactive mutant (Figure 1A). Extracting ions from the peptides that contain R35 from both proteins confirmed the absence of methylation on this residue within the KLA mutant (Figure 1B). The observation that PRMT6-WT was methylated in the absence of added substrate suggested that most of the active protein was automethylated during bacterial expression and also provided an explanation for the low levels of automethylation observed in previous studies [17]. We also confirmed that R35 is methylated *in vivo* by conducting an *in vivo* methylation assay (Figure 1E).

**Figure 2:**
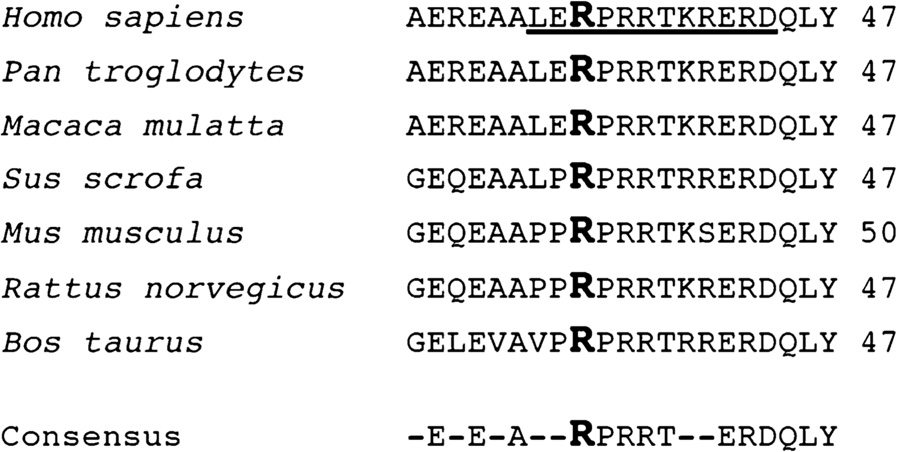
**The R35 residue is conserved in evolution.** Sequence alignment of PRMT6 proteins from various organisms showing conservation of the arginine residue at position 35 (*H*. *sapiens*). The consensus sequence was produced using ClustalW2 (http://www.ebi.ac.uk/Tools/msa/clustalw2/). R35 is bolded and the underlined text refers to the arginine rich motif.

Alignment studies show that the R35, R37, and R38 residues are well conserved among mammals e.g. *Homo sapiens*, *Pan troglodytes*, *Macaca mulatta*, *Sus scrofa*, *Mus musculus*, *Rattus norvegicus*, and *Bos Taurus* (Figure 2), but the region is not conserved in some other organisms, e.g. *Arabidopsis thaliana* and *Danio rerio* (data not shown), suggesting that its emergence followed the divergence that occurred between these branches in evolution. R29 is poorly evolutionary conserved.

### PRMT6 is automethylated at R35

**Figure 3:**
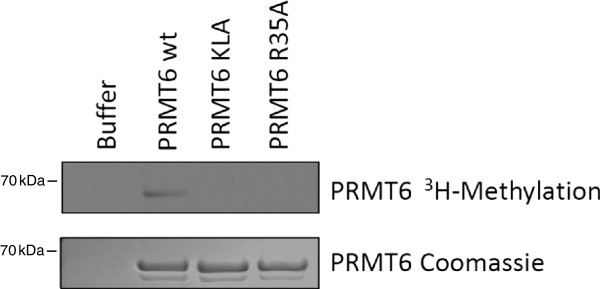
**PRMT6**-**R35A does not automethylate.** Cell-free automethylation assays were performed using the indicated PRMT6 recombinant proteins in the presence of ^3^H-SAM. Autoradiography (upper) and Coomassie staining (lower) are shown. ^3^H-SAM was contained in all wells. The first lane contains methylation buffer only, without the presence of PRMT6. The other lanes contain the indicated recombinant proteins. This experiment was performed three times with similar results being obtained each time; a representative result is shown.

**Figure 4:**
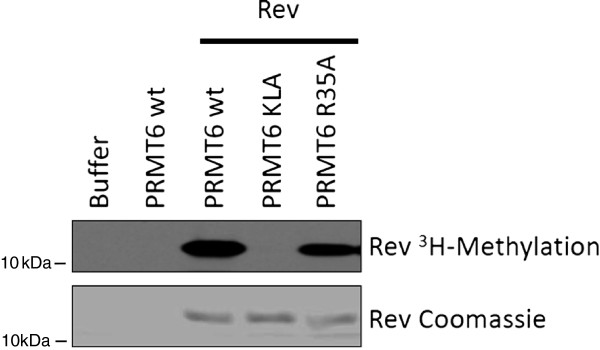
**PRMT6**-**R35A is catalytically active.** A PRMT6 cell-free methylation assay was performed with HIV-1 Rev protein, a known substrate of PRMT6. The upper frame represents the autoradiograph of Rev methylation by PRMT6. The lower frame represents a Coomassie stained gel of Rev. This experiment was performed three times with similar results obtained each time; a representative result is shown.

To characterize the role of automethylation in the activity of PRMT6, the central residue R35 was mutated to an alanine residue (PRMT6-R35A) and automethylation was measured *in vitro* in the presence of ^3^H-SAM (Figure 3). Mutant PRMT6-KLA was used as a negative control. Weak but consistent automethylation was measured for the wild-type recombinant PRMT6 protein but not for either KLA or R35A, suggesting that R35 plays a critical role in automethylation. Therefore our study focused on the role of this residue. In a similar experiment, PRMT6-R35A was shown to be able to methylate the purified HIV-1 Rev protein *in vitro*, indicating that this mutated derivative protein retains its ability to arginine methylate other proteins but not itself (Figure 4). In the same experiment, PRMT6-KLA was, as expected, completely unable to methylate Rev. Thus, R35 is a PRMT6 primary automethylation site and automethylation may not be essential for the methylation of HIV-1 Rev. We performed similar *in vitro* methylation experiments using HIV-1 Tat as a substrate and found that PRMT6-R35A maintains methylation activity (data not shown).

### PRMT6 automethylation regulates its stability

**Figure 5:**
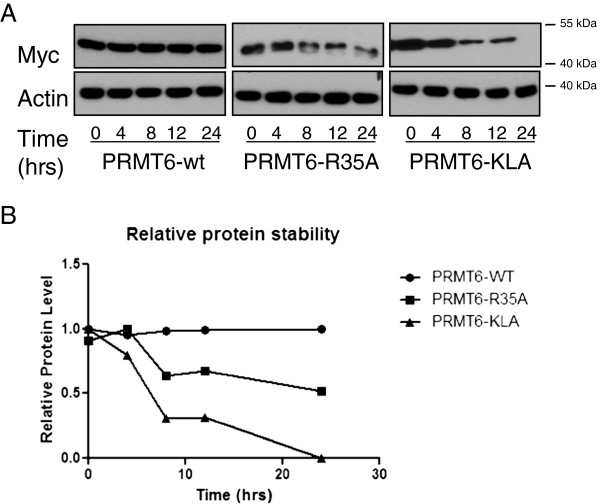
**PRMT6**-**R35A is less stable than PRMT6**-**WT. (A)** Western blots for Myc-PRMT6-WT, PRMT6-R35A and PRMT6-KLA, following treatment with CHX (upper panels). Actin was used as a loading control (lower panels). 15 μg of protein from whole cell extract were loaded into each well. **(B)** Densitometric analysis of Myc-PRMT6-WT and -R35A degradation over time following the addition of CHX. Myc expression was normalized to levels of actin. Each experiment was performed three times with similar results obtained each time; a representative blot is shown.

We next measured the stability of the wild-type, KLA, and R35A PRMT6 proteins (Figure 5). Plasmids coding for the various forms of myc-tagged PRMT6 were transfected into HeLa cells, and, at 24 h after transfection, cycloheximide (CHX) was added to culture supernatants. Since CHX inhibits protein synthesis, it can be used to study protein degradation over time in studies in which expression levels of PRMT6 protein are measured by Western-blot. The results show that PRMT6 is very stable (Figure 5A, first panel), but that the mutant R35A protein and the catalytically inactive KLA form of PRMT6 were less stable, with their expression levels decreasing over time. Densitometric quantification of the Western-blot confirmed that the expression levels of both mutant proteins decreased faster than the WT protein following the addition of CHX (Figure 5B). Thus, PRMT6 automethylation is important for its stability.

### PRMT6 automethylation is required for inhibition of HIV-1 replication

**Figure 6:**
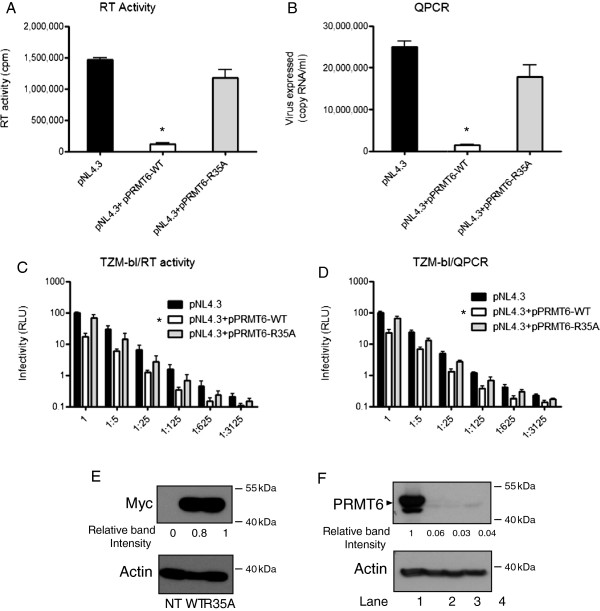
**PRMT6 automethylation is necessary for its HIV-1 restriction activity.** The indicated forms of PRMT6 plasmid were co-transfected with HIV-1 pNL4.3 proviral DNA into 293T cells. At 48 hours after transfection, cell culture fluids containing virus were collected, quantified by QPCR **(A)** or by HIV RT activity assay **(B)**, and titrated onto TZM-bl cells **(C, D)**. The amount of transfected PRMT6 expression, i.e. not protein stability, was quantified by Western blots with anti-Myc and anti-actin antibodies followed by densitometric analysis normalized against actin **(E)**. Endogenous levels of PRMT6 in HeLa cells (lane 2), TZM-bl cells (lane 3) and 293T cells (lane 4) were compared to transfected PRMT6 in HeLa cells (lane 1) **(F)**. 15 μg of protein from whole cell extracts were loaded into each well used for analysis in the Western blots in **(E)** and **(F)**. Actin was used as a loading control. Infectivity was measured by luciferase assay at 48 hours after infection. The control experiment was transfection of pNL4.3 with an empty plasmid (referred to on the figure as pNL4.3 only). Each experiment was performed in triplicate on three separate occasions, with similar results being obtained each time. T-tests were performed for both **(A)** and **(B)**, showing that only PRMT6-WT was significantly different than the control *: p < 0.05. Two-way ANOVA was performed for **(C)** and **(D)**, showing that only PRMT6-WT statistically inhibited HIV replication *: p < 0.001.

We have shown that the expression level of PRMT6 is important for inhibition of HIV-1 replication, and now wished to assess the impact of the R35A mutation on this activity (Figure 6). Proviral DNA coding for pNL4-3 virus was co-transfected into 293T cells together with either a control empty plasmid or plasmids coding for PRMT6-WT or R35A mutant proteins. The resulting viruses were quantified by RT activity assay and by QPCR (Figure 6A and B) and their infectiousness was tested in TZM-bl reporter cells by normalizing the amount of infecting virus for either RT activity or viral RNA (Figure 6C and D). Since PRMT6-WT and R35A display differences in their stability (Figure 5), we verified the proper expression of these proteins in these experiments by measuring PRMT6 protein expression at 24 hours post transfection by Western blot (Figure 6E). Densitometric quantification indicated that PRMT6-WT expression levels were 20% lower than those of PRMT6-R35A in these experiments, measures that are independent from differences in stability. The data show that expression of PRMT6-WT decreased HIV-1 infectivity, but that the R35A mutation restored viral infectivity to levels similar to those of virus grown in the absence of PRMT6. This indicates that prevention of PRMT6 automethylation results in a significant decrease in PRMT6 anti-HIV activity of approximately 90% (Figure 6).

## Discussion

Although PRMT6 is known to be able to restrict the activity of the HIV-1 Tat, NC, and Rev proteins as well as viral replication, the role of PRMT6 automethylation in this process has been unknown. Additionally, the identity of the residue(s) targeted for automethylation has remained elusive. Here, we have shown that R29, R35, and R37 of PRMT6 are auto-dimethylated, as demonstrated in a cell-free reaction. PRMT6 automethylation sites were confirmed *in vivo*. Mutating R35 to an alanine resulted in the inhibition of PRMT6 automethylation. We have further shown that automethylation is required for PRMT6 protein stability and its anti-HIV-1 activity. This is important as several inhibitors of PRMT proteins are currently being developed for cancer therapy and could also have potential for treatment of HIV infection [31].

Major host restriction factors (HRFs) that control HIV replication in specific cell types include TRIM5α, APOBEC3G, tetherin and SAMHD1. Some of these HRFs are regulated by post-translational modifications, i.e. phosphorylation and ubiquitination for APOBEC3G [32-34]. The current study reinforces the fact that arginine methylation can play a role in regulation of anti-HIV activity, and, in addition, is the first to identify R35 as a major residue targeted for automethylation within PRMT6. Finally, preventing PRMT6 automethylation by introducing the R35A mutation antagonized the antiretroviral effects of this protein, demonstrating that PRMT6-mediated anti-HIV activity is both specific and that this activity is regulated *in vivo*.

To identify potential methylated arginines, we performed mass spectrometric analysis on recombinant PRMT6-WT and PRMT6-KLA. These studies led to the identification of three arginine residues, i.e. R29, R35, and R37 in the N-terminal region of PRMT6 that are specifically methylated in the WT protein but not in the KLA mutant. This indicated that these residues are modified through automethylation. Additionally, we show here that PRMT6 is automethylated in the absence of added SAM, suggesting that most of PRMT6 was automethylated during bacterial expression. This may provide an explanation for the low levels of PRMT6 *in vitro* automethylation reported previously [17]. Other related enzymes including PRMT1, CARM1 and PRMT8 have all been described as possessing automethylation activity [28,35,36]. In similar fashion to PRMT6, as shown here, CARM1 automethylation has been shown to be dispensable for its enzymatic activity *in vitro*, but can still affect the ability of the enzyme to activate transcription and to regulate pre-mRNA splicing [28].

Following CHX treatment, both the methyltransferase deficient PRMT6-KLA mutant and the PRMT6 R35A mutant displayed considerably less stability than did WT PRMT6. Arginine methylation has been linked to protein stability in several previous studies. For example, PRMT1 arginine methylation of axin increases the stability of the latter by blocking ubiquitination [30]. PRMT3 methylation of the 40S ribosomal protein S2 (rpS2) also increases protein stability by reducing rpS2 ubiquitination [37]. Importantly, PRMT6 methylation has been shown to increase the half-life of the HIV-1 Tat protein without changing its ubiquitination pattern [29]. We are currently trying to determine whether the enhanced degradation of PRMT6-KLA and PRMT-R35A may also be due to ubiquitination.

Our findings further show that methylated PRMT6 is able to restrict HIV-1 replication whereas the ability of R35A-PRMT6 to play this role is diminished by as much as 90% (Figure 6A and B). Since PRMT6-R35A has intact catalytic activity *in vitro*, we believe that its diminished restriction activity may be due to a reduced availability of mutated PRMT6 within the cell, as a result of its poor stability. When viral input was normalized by QPCR or RT activity, WT PRMT6 restriction was carried over to a second cycle of infection, as shown in a TZM-bl infectivity assay (Figure 6C and D). This suggests that PRMT6 methylation affects both initial virus production as well as subsequent rounds of replication.

A recent study reported that arginine methylation of antigenic peptides displayed on human leukocyte antigens (HLA) resulted in specific recognition by the immune system and elicited a T-cell response [38]. It would be interesting to determine whether PRMT6 methylated HIV-1 proteins can trigger an equivalent response, as this might represent a means of eliciting an anti-HIV-1 immunological response. Although it is clear that arginine methylation plays an important regulatory role in protein stability, further investigation is needed to clarify the role that PRMTs play in regard to proteasomal degradation pathways.

## Conclusion

PRMT6 is automethylated at position R35 and this event plays an important role in regard to the stability of this protein. Due to problems of degradation of non-automethylated PRMT6, the ability of the non-automethylated protein to restrict viral replication is greatly reduced.

## Methods

### Reagents

Recombinant glutathione-S-transferase (GST)-tagged wild-type PRMT6 (GST-PRMT6-WT), methyltransferase inactive V86K/D88A PRMT6 (GST-PRMT6-KLA) and histidine-tagged HIV-1 Rev protein (His-Rev) were prepared as described previously [23]. Myc-tagged wild-type PRMT6 (myc-PRMT6-WT) and inactive Myc-tagged methyltransferase V86K/D88A PRMT6 (Myc-PRMT6-KLA) have also been described previously [22]. GST- and Myc-tagged PRMT6-R35A mutants were generated using the QuikChange Site-Directed Mutagenesis Kit (Stratagene, La Jolla, CA, USA) through use of a 5′-GCGGCCCTGGAGGCACCCCGGAGGAC-3′ forward primer and a 5′-GTCCTCCGGGGTGCCTCCAGGGCCGC-3′ reverse primer (Invitrogen). To quantify viral genomic RNA, quantitative real-time polymerase chain reaction (QPCR) primers and probe used were 5′-CCGTCTGTTGTGTGACTCTGG- 3′ forward Primer, 5′-GAGTCCTGCGTCGAGAGATCT-3′ reverse primer and 5′ FAM- TCTAGCAGTGGCGCCCGAACAGG- TAMRA-3′ probe (Invitrogen) [39]. Anti-PRMT6 rabbit polyclonal antibody was purchased from Imgenex and anti-Myc mouse monoclonal antibody was purchased from Invitrogen. Anti-actin mouse monoclonal antibody was purchased from MP Biomedicals.

The following reagents were obtained through the NIH AIDS Research and Reference Reagent Program, Division of AIDS, NIAID, NIH: TZM-bl from Dr. John C. Kappes, Dr. Xiaoyun Wu and Tranzyme Inc and pNL4-3 from Dr. Malcolm Martin [40].

### Liquid chromatography-mass spectrometry analysis

Recombinant GST-PRMT6-WT and GST-PRMT6-KLA (5 μg each) were incubated with 25 μM S-adenosylmethionine (Sigma) in 25 mM Tris–HCl (pH 7.4) for 3 hours at 37°C in a final volume of 30 μl. The reactions were stopped by adding 6 μl of 5× Läemmli buffer, followed by boiling for 5 minutes and centrifugation at 16,000 g for 2 minutes. The samples were then run on 10% SDS polyacrylamide gels. Bands corresponding to GST-PRMT6-WT and GST-PRMT6-KLA were cut out, processed, and analysed by liquid chromatography-tandem mass spectrometry (LC-MS/MS).

Prior to HPLC the polyacrylamide gel plug containing GST-PRMT6 was subjected to in-gel tryptic digestion for protein ID and identification of the dimethylated arginines. Briefly the polyacrylamide gel plug was destained with 25 mM ammonium bicarbonate (NH_4_HCO_3_) and 50% acetonitrile (ACN). The gel particle was then incubated with 100% ACN. Reduction was accomplished with 10 mM DTT and alkylation with 55 mM iodoacetamide. After washing with 50 mM NH_4_HCO_3,_ the gel particles were shrunk with 100% ACN and dried down in a SpeedVac. In order to detect methylated peptides, samples underwent partial in-gel digestion with 1.6 μg of Trypsin (Sigma), an enzyme that does not digest the C-terminus of methylated arginine. Gel particles were vortexed and sonicated in 5% formic acid (FA)/50% ACN to extract sample from the gel. The volume of the sample was reduced to 15 μl in the SpeedVac and cleaned using a C18 ZipTip (Millipore).

The samples were dried down in a SpeedVac apparatus and resolubilized in 50 μl of acetonitrile (ACN) 5% /formic acid (FA) 0.1%. 2 μl of each sample were directly injected onto a C18 analytical column (75 μm i.d. × 100 mm) using the Proxeon EASY nLC system. A 21-min gradient was used to elute peptides at a flow rate of 300 nl/min. The gradient started at 3% acetonitrile/0.2% formic acid and a linear gradient to 35% acetonitrile/0.2% formic acid was achieved in 13 min, then ramped up to 92% acetonitrile 0.2% formic acid after 2 minutes.

The liquid chromatography (LC) system was coupled to a LTQ-Orbitrap mass spectrometer (MS) (Thermo Fisher). A full MS spectrum was collected at the level of the Orbitrap (FT-MS); then, the ten most abundant multiply charged ions (threshold > 5000 counts) were selected for MS/MS sequencing at the level of the linear trap and stored in an exclusion list for 30 seconds. Tandem MS experiments were performed using a collision-induced dissociation set at 35% with activation time of 10 msec. The data were processed using Proteome Discoverer (version 1.3) running the SEQUEST search engine. Database searching against a FASTA file containing 136 sequences (mostly bacterial, yeast and mammalian contaminants) including PRMT6 WT and mutant sequences was performed allowing differential modification on cysteine residues (carbamidomethylation: +58), methionine (oxidation: + 16) and arginine (dimethylation: +28). MS/MS spectra were searched for protein tryptic digests allowing a maximum of two missed cleavage sites per peptide. Only peptides with Xcorr values greater than 3.2, equivalent to a false discovery rate lower than 1%, were retained to assess coverage of PRMT6 (WT and Mutant) and dimethylation sites.

### In vitro methylation assay

Recombinant GST-tagged PRMT6 proteins (3–4 μg) were incubated with 1–2 μg of recombinant histidine-tagged Rev with 0.55 μCi of [methyl-3H]-S- adenosyl-L-methionine (Perkin Elmer life sciences) and 25 mM Tris–HCl (pH 7.4) for 3 hours at 37°C in a final volume of 25 μl. Reactions were stopped by adding 5 μl of 5 × Läemmli buffer followed by boiling for 5 minutes and centrifugation at 16,000 g for 2 minutes. Samples were loaded onto 10% SDS polyacrylamide gels. Gels were stained with Coomassie brilliant blue R-250 solution (Bio-Rad Laboratories) and, after destaining, soaked in 1× ENH^3^ANCE (Perkin Elmer life sciences) for 45 minutes. Gels were then dried and exposed on HyBlot CL autoradiography film (Denville Scientific) for 1 to 3 days. Gels and films were quantified with the ImageJ software [41]. Automethylation assays were similarly performed; however, the incubation period was increased to between 14 and 21 days for reasons of enhanced sensitivity.

### In vivo methylation assay

HeLa cells were transfected with Lipofectamine 2000 reagent according to the manufacturer’s guidelines (Invitrogen) with 1 μg myc tagged-PRMT6 WT DNA. At 24 hr after transfection, S-adenosyl-L-methionine (New England Biolabs) was added to the cells. Cells were collected at 48 h post transfection and lysed with RIPA buffer, and 30 μl of lysate was then incubated with myc-conjugated agarose beads (Sigma). After several washes, beads were boiled in the presence of Laemmli buffer and centrifuged. The supernatant containing the immunoprecipitated protein was run on an SDS-PAGE gel. The appropriate band was processed for LC-MS/MS as described above.

### Cell culture

HeLa, 293T and TZM-bl cells were all cultured in Dulbecco modified Eagle medium (DMEM) (Gibco), supplemented with 10% fetal bovine serum, 50 IU of penicillin/ml, 50 μg of streptomycin/ml, and 2 mM L-glutamine at 37°C in 5% CO_2_.

### Cycloheximide treatment and immunoblotting

HeLa cells were transfected with Lipofectamine 2000 reagent according to the manufacturer’s guidelines (Invitrogen) with 1 μg myc tagged-PRMT6 WT, KLA or R35A plasmid. At 24 hr after transfection, the cells were treated with 100 μg/ml of cycloheximide (Sigma) and collected after 0, 2, 4, 6 and 8 hr. Cells were lysed with RIPA buffer containing protease inhibitor cocktail (Sigma). Protein concentrations of whole cell extracts were quantified using Bradford assays (5× BioRad Protein assay). 15 μg of protein was boiled with Läemmli buffer for 5 minutes and samples were loaded onto a 10% SDS polyacrylamide gel. Western blots were performed on a PVDF membrane (BioRad). Bands were quantification with ImageJ (http://rsb.info.nih.gov/ij/).

### Viral production and quantification

293T cells were transfected as described above. Cells (3 × 10^6^) were co-tranfected with 4 μg of pNL4.3 and 4 μg of myc-PRMT6 plasmids; at 48 hours post transfection, culture supernatants were collected, centrifuged at 1,200 rpm and passed through a 0.45 μm filter. Viral particles in the supernatants were quantified by quantitative real-time PCR (QPCR) as described previously [42] and by HIV reverse transcriptase (RT) activity assay, also previously described [43]. The same supernatants were then used to measure infectivity in TZM-bl, as described below. Experiments were repeated 3 separate times and data were analysed using GraphPad Prism software.

### Infectivity assay

Viruses produced in 293T cells, as described above, in the presence of myc-PRMT6 plasmids were normalized on the basis of QPCR or HIV RT activity and were 5-fold titrated onto TZM-bl luciferase reporter cells and incubated for 48 hours. TZM-bl cells were lysed using the luciferase assay system (Promega) and luminescence was read with a MicroBeta Trilux Luminescence counter over a period of 1 second (Perkin Elmer). Experiments were repeated 3 times and data were analyzed using GraphPad Prism software.

## Competing interests

The authors declare that they have no competing interests.

## Authors’ contributions

Conceived and designed the experiments: DNS, MAW. Performed the experiments: DNS, TM, AS, JPF. Analyzed the data: MAW, DNS, TM, PKQ, JPF. Wrote the paper: DNS, TM, MAW. All authors approved the submission of the manuscript. This work was largely performed by DNS in partial fulfilment of the requirements of a PhD degree, Faculty of Graduate Studies and Research, McGill University, Montreal, Quebec, Canada.
